# Supplementary material for: Sustainable and Printable Nanocellulose-Based Ionogels as Gel Polymer Electrolytes for Supercapacitors
Source: Nanomaterials (Basel). 2022 Jan 15;12(2):273. doi: 10.3390/nano12020273 (PMC8780157; doi:10.3390/nano12020273)
Supplement: Supplementary file 1 [file nanomaterials-12-00273-s001.zip › nanomaterials-1545230-supplementary.pdf]

Supplementary Materials

Sustainable and printable nanocellulose-based ionogels as gel polymer electrolytes for supercapacitors

Rosa M. González-Gil [1,2,†], Mateu Borràs [3,4,†], Aiman Chbani [3], Tiffany Abitbol [5], Andreas Fall [5], Christian Aulin [5,6], Christophe Aucher [3,*] and Sandra Martínez-Crespiera [1,*]

1 Applied Chemistry and Materials, ARTS Department, Leitat Technological Center, C/Pallars, 186-179,
08005 Barcelona, Spain; rosamaria.gonzalez@icn2.cat
2 Novel Energy-Oriented Materials Group, Catalan Institute of Nanoscience and Nanotechnology, ICN2 (CSIC-BIST), Edifici ICN2, Campus UAB, 08193 Barcelona, Spain
3 Energy and Engineering, ARTS Department, Leitat Technological Center, C/de la Innovació, 2,
08225, Barcelona, Spain; mateu@bioo.tech (M.B.); achbani@leitat.org (A.C.)
4 Arkyne Technologies SL (Bioo), C/de la Tecnología, 17, 08840, Barcelona, Spain
5 Bioeconomy and Health, RISE Research Institutes of Sweden, Drottning Kristinas väg 61,
114 28 Stock-holm, Sweden; tiffany.abitbol@ri.se (T.A.); andreas.fall@ri.se (A.F.); Christian.aulin@Holmen.com (C.A.)
6 Holmen Iggesund, 825 80 Iggesund, Sweden
* Correspondence: caucher@leitat.org (C.A.); sandramartinez@leitat.org (S.M.-C.); Tel.: +34 93 788 23 00 (S.M.-C.)
† These authors contributed equally to this work.

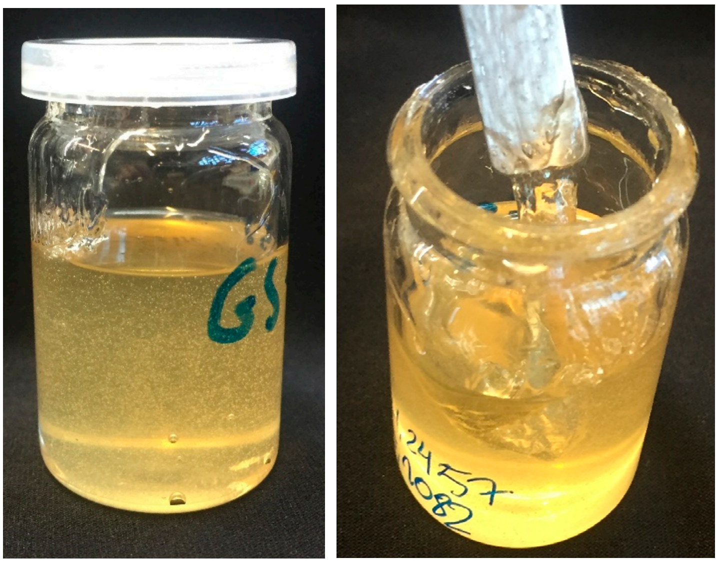

Figure S1: Final aspect of the as-prepared ionogels pictures**.**

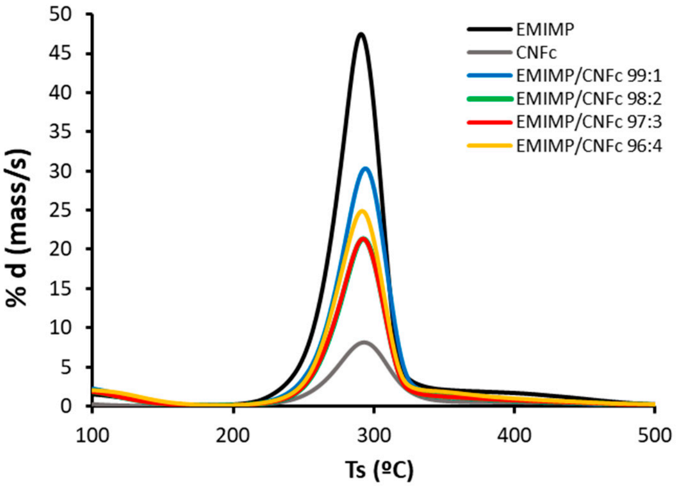

Figure S2: Differencial scanning calorimetry thermogram (DSC) of the EMIMP/CNFc mixtures (99:1, 98:2, 97:3 and 96:4). Pristine CNFc and EMIMP were added for comparison purposes.

	**EMIMP/NC ratio**	**T decomposition (ºC)**
EMIMP	100:0	290,0
CNFc	0:100	294,2
EMIMP/CNFc 99:1	99:1	292,0
EMIMP/CNFc 98:2	98:2	292,3
EMIMP/CNFc 97:3	97:3	292,7
EMIMP/CNFc 96:4	96:4	293,0

Table S1: Temperature decomposition for each EMIMP/CNFc mixtures (99:1, 98:2, 97:3 and 96:4) mixtures, obtained from differential scanning calorimetry thermograms (DSC). Pristine CNFc and EMIMP temperatures are also added.

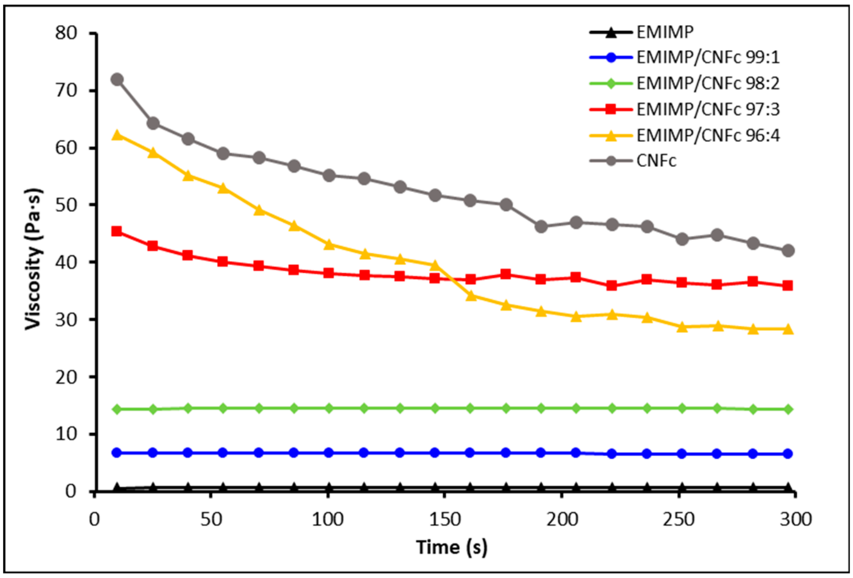

Figure S3: Rheological results at room temperature vs. time of the EMIMP/CNFc mixtures (99:1, 98:2, 97:3 and 96:4). Pristine CNFc and EMIMP are added for comparison purposes.

Electrolyte	i (mA/cm²)	C_e (F g^{-1})	ESR (Ω cm^{-2})	E_{max} (Wh kg^{-1})	P_{max} (W kg^{-1})
EMIMP	0.5	109±3	15±1	15.2±0.5	5872.2±490.3
	1	99± 4	14.7± 0.7	13.5±0.7	5924.9±485.5
	2	75±4	14.6±0.7	10.2±0.7	6003.3±475.9
	5	35±4	14.7±0.5	5.5±1.4	5974.7±473.5
	10	10±3	15.4±0.6	1.5±0.9	5908.8±764.1
EMIMP99:1	0.5	159±7	11±2	22.7±1.2	9536.2±2219.8
	1	132± 4	8.2± 0.8	18.7±0.7	11,280.3±2169.9
	2	105±3	7.7±0.8	14.4±0.5	11,796.8±2093.7
	5	59±6	8.4±0.6	7.9±1.1	11,155.5±2209.7
	10	29±7	7.9±0.8	3.6±1.1	10,934±1987
EMIMP98:2	0.5	174±9	11±4	24.1±1.6	7618.5±2107.4
	1	156± 11	10± 2	21.5±1.8	8132.8±2364.1
	2	127±15	9±2	17.8±2.5	8311.8±2479.6
	5	73±21	10±2	10.3±3.6	8107.3±2487.9
	10	30±16	10±2	4.2±2.7	7695±2462
EMIMP97:3	0.5	172±6	12±1	24.0±1.2	7677.7±938
	1	160± 6	10.2± 0.7	22.3±1.2	8841.9±790.6
	2	139±9	9.7±0.5	19.3±1.8	9330±659
	5	93±12	9.6±0.3	12.9±2.4	9332.7±350.2
	10	44±9	10.2±0.2	6.2±1.8	8823±525
EMIMP96:4	0.5	165±4	7.4±0.5	22.5±0.8	12,717±986
	1	152± 5	5.6± 0.6	20.8±0.9	16,023±1524
	2	130±8	5.3±0.3	18.1±1.3	16,729.1±1723.5
	5	91±12	5.4±0.2	13.3±2.0	16,429.1±1767.6
	10	56±11	5.7±0.3	8.5±1.8	15,463.4±1415.7

Table S2: Summary of the key performance parameters on different current density such as specific capacitance, ESR, E_{max} and P_{max}

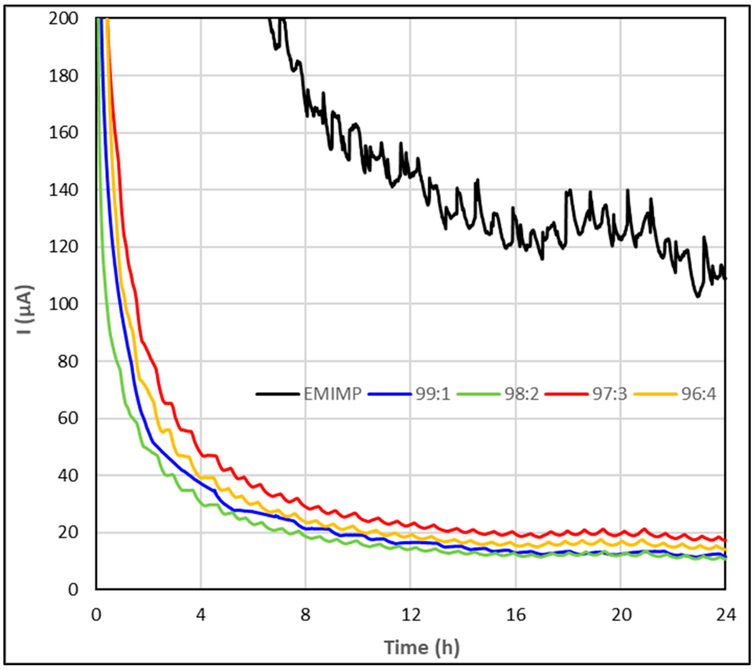

Figure S4: Current (μA) vs. time for supercapacitors with EMIMP and EMIMP/CNFc (99:1, 98:2, 97:3 and 96:4) as electrolytes for a period of 24h

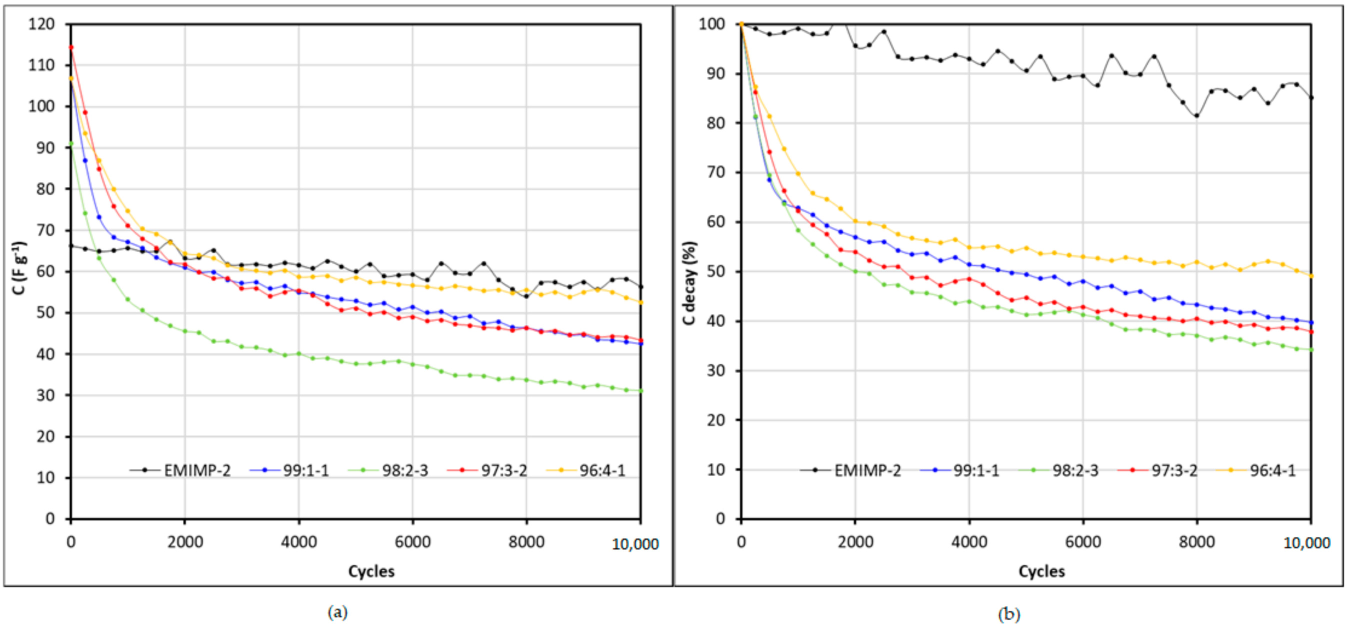

Figure S5: The capacity variation with the cycles and for supercapacitors with EMIMP and EMIMP/CNFc (99:1, 98:2, 97:3 and 96:4) as electrolytes.

Electrolyte	i (mA/cm^2)	C_e ($F\ g^{-1}$)	ESR ($\Omega\ cm^{-2}$)
EMIMP/CMC	0.5	153±3	11.8±1
	1	123± 4	10.6± 0.7
	2	96±4	10.3±0.7
	5	54±4	10.4±0.5
	10	23±3	10.6±0.6
EMIMP99:1	0.5	159±7	11±2
	1	132± 4	8.2± 0.8
	2	105±3	7.7±0.8
	5	59±6	8.4±0.6
	10	29±7	7.9±0.8

Table S3: Summary of the key performance parameters on different current density such as specific capacitance and ESR of EMIMP/CMC vs EMIMP/CNFc 99:1

	Scan rate ($mV\ s^{-1}$)	C_e ($F\ g^{-1}$)
EMIMP	10	2.4
	50	0.6
EMIMP99:1	10	6.9
	50	1.1
EMIMP98:2	10	8.1
	50	1.1
EMIMP97:3	10	8.3
	50	1.2
EMIMP96:4	10	7.3
	50	1.1

Table S4: Summary of specific capacitance of EMIMP and ionogels with different ratio of CNFc at 10 $mV\ s^{-1}$ and 50 $mV\ s^{-1}$

scan rate for CV

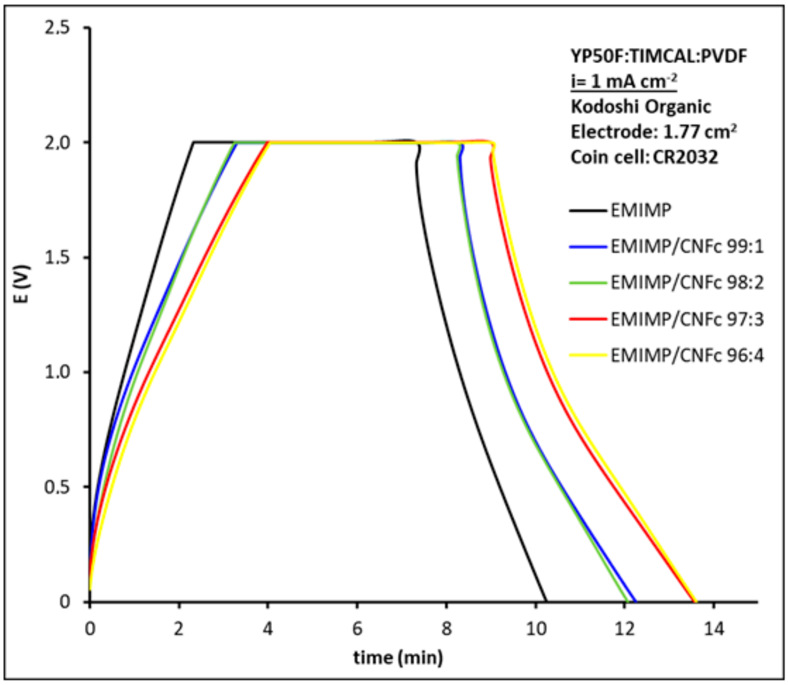

Figure S6: Galvanostatic charge-discharge curve of EMIMP and ionogels with different ratio of CNFc at i= 1 mA cm^{-2}

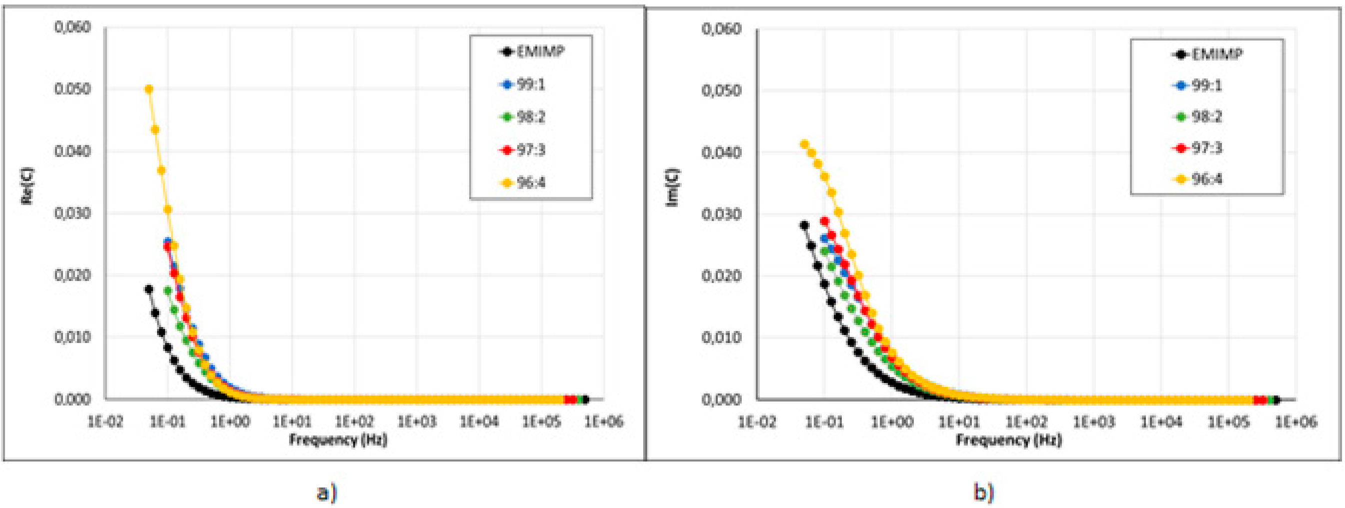

Figure S7: a) Real part of the capacitance vs Frequency and b) Imaginary part of the capacitance versus Frequencies of EMIMP and ionogels with different ratio of CNFc
